# Chloramphenicol Selection of IS10 Transposition in the *cat* Promoter Region of Widely Used Cloning Vectors

**DOI:** 10.1371/journal.pone.0138615

**Published:** 2015-09-16

**Authors:** Coral González-Prieto, Leticia Agúndez, Matxalen Llosa

**Affiliations:** 1 Departamento de Biología Molecular (Universidad de Cantabria) and Instituto de Biomedicina y Biotecnología de Cantabria (UC-CSIC-SODERCAN), Santander, Spain; 2 Department of Genetics, University College London, Institute of Ophthalmology, London, United Kingdom; University of Minnesota, UNITED STATES

## Abstract

The widely used pSU8 family of cloning vectors is based on a p15A replicon and a chloramphenicol acetyltransferase (*cat*) gene conferring chloramphenicol resistance. We frequently observed an increase in the size of plasmids derived from these vectors. Analysis of the bigger molecular species shows that they have an IS10 copy inserted at a specific site between the promoter and the *cat* open reading frame. Promoter activity from both ends of IS10 has been reported, suggesting that the insertion events could lead to higher CAT production. Insertions were observed in certain constructions containing inserts that could lead to plasmid instability. To test the possibility that IS10 insertions were selected as a response to chloramphenicol selection, we have grown these constructs in the presence of different amounts of antibiotic and we observed that insertions arise promptly under higher chloramphenicol selective pressure. IS10 is present in many *E*. *coli* laboratory strains, so the possibility of insertion in constructions involving *cat*-containing vectors should be taken into account. Using lower chloramphenicol concentrations could solve this problem.

## Introduction

Universal cloning vectors offer a series of advantages to facilitate their manipulation, which include their small size, presence of multiple cloning sites, selection markers, and high copy number. The primarily used cloning vectors are associated with the pMB1 replicon. They carry most often the beta-lactamase gene from Tn*3*, originally present in the historic vector pBR322 (GeneBank Accession number J01749; [1]), allowing plasmid selection by resistance to ampicillin. Cloning vectors based on the p15A replicon are the common alternative to pMB1-based vectors. They have a moderate copy number and therefore they are a better option when potentially toxic genes are to be cloned. Most of these vectors encode the *cat* gene from *Tn9*, as in original vector pACYC184 (GeneBank Accession number X06403; [2]), conferring chloramphenicol resistance (Cm^R^). The most widely used family of p15A-derived cloning vectors is the pSU8 family [3, 4], which was designed to allow coexistence with a pMB1-Ap^R^ vector in the same host. The widespread use of this family of vectors can be estimated by the hundreds of cites of their original article (ISI Web of Science). pSW family of vectors are conditionally replicating plasmids based on the IncX *oriV* origin of replication which are dependent on the *pir*-encoded protein [5]. These vectors are also Cm^R^ and carry the same *cat* gene as the pSU8 family.

IS10R, the right module of the bacterial transposon Tn10, can act as an individual insertion sequence since it codifies a transposase protein [6] with a transposition frequency of 10^−4^ per cell per bacterial generation [7]. Although the originally sequenced genomes of both *Salmonella* and *E*. *coli* strains [8, 9] did not show any copy of IS10, there have been several reports of the presence of IS10 elements in different *Salmonella* and *E coli* strains, some as widely used as *E*. *coli* JM109, DH5α, DH10B or XL-2 Blue [10, 11], and IS10 transposition events are frequently detected in *E*. *coli* [12]. Thus, we can assume that IS10 copies are present in the genomes of many commonly used *E*. *coli* K-12 laboratory strains. In fact, there have been previous reports on cloning artefacts due to IS10 transposition from *E*. *coli* genome to a plasmid. IS10 insertion has been reported to take place frequently during cloning in pUC19 vector [13]. Kovarik and co-workers performed a database search that concluded that IS10 was inserted into numerous eukaryotic clones [10].

Bacterial conjugation is a process of DNA transfer between bacteria [14]. The conjugation machinery, usually encoded by conjugative plasmids which are self-transmissible, includes a number of proteins required for DNA processing and secretion, plus a coupling protein linking the secretion machinery to the transferred DNA. The transfer process starts and ends at a DNA segment named the origin of transfer (*oriT*), the only site required in *cis* for conjugation. A number of conjugative proteins bind to this site, such as the conjugative relaxase, which cuts the *oriT* and reseals it after transfer. In conjugative plasmid R388, the coupling protein is named TrwB, and the relaxase, TrwC.

In spite of the successful use of pSU cloning vectors for more than 20 years, we recently detected an apparent genetic instability in several constructs based on pSU and pSW vectors that harbored different elements of conjugative machineries. Analysis of this phenomenon led us to discover an insertional target sequence for IS10 in the pSU backbone, which may lead to increased *cat* expression. Insertion events were selected under high Cm concentrations only for certain plasmid constructs that may cause toxicity or plasmid instability. This phenomenon, which may be overcome by the use of mild selection conditions, should be taken into account by the many researchers worldwide using this popular family of cloning vectors.

## Results

### An insertion target for IS10 in the pSU8 family of vectors

**Fig 1:**
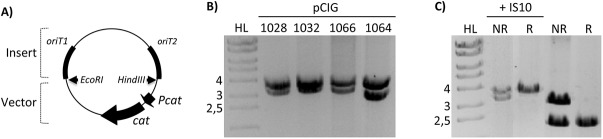
Restriction analysis of recombination substrate plasmids with EcoRI and HindIII restriction enzymes. **A)** Scheme of the recombination substrate plasmids. EcoRI and HindIII restriction sites were used to clone the insert containing the two *oriT* copies, which are shown as black rectangles. The vector-encoded *cat* gene is indicated as a black arrow. *Pcat*, *cat* promoter. **B)** In not recombined substrate plasmids, we expected a band of 2.3 kb for vector pSU19 and a band of 3.2 kb for the insert containing both *oriTs* (3.0 kb for pCIG1064, due to the smaller size of the cloned F *oriT*s). The band of the vector is not detected; instead a band of about 3.5 kb is visible. **C)** The expected bands are seen in pCIG1028 when IS10 was not present (2.3 kb + 3.2 kb bands for non-recombined and 2.3 kb for recombined plasmid, as the band of 0.4 kb corresponding to the unique *oriT* is not visible). When IS10 is present, the vector band increases up to around 3.5 kb. HL, Hyperladder I (Bioline), with the size of relevant bands indicated in kb on the left. NR, not recombined. R, recombined.

We routinely use the pSU8 family of vectors in *E*. *coli*, and we observed several times that the restriction pattern of different plasmid constructions based on these vectors was not the expected one. An increase in size of about 1 kb was observed. Fig 1A shows a map of some of the plasmids used in this work where this phenomenon can be observed. The restriction analysis is shown in Fig 1B. The band corresponding to the insertion fragment was present in all of them. However, the band corresponding to pSU19 vector that should be 2.3 kb in size was not visible. Instead, a band of approximately 3.5 kb was detected.

This phenomenon was observed only in some constructs. In particular, plasmids shown in Fig 1 encode two conjugative *oriTs*. It has been reported that certain relaxases can catalyse *oriT*-specific recombination, leading to the loss of the DNA segment present between two direct repeats of their cognate *oriT* [15–18]. These plasmids were used as recombination substrates, and differ only in the source plasmid of the *oriT* copies: pCIG1028 (R388), pCIG1032 (pKM101/R388), pCIG1066 (pKM101) and pCIG1064 (F) [15]. Fig 1C shows restriction analysis of pCIG1028 DNA in the four different forms found during its manipulation: before and after *oriT*-specific recombination, both with and without the increase in size. Similar results were found when analysing the rest of the plasmids mentioned above.

In addition, we observed this same phenomenon in plasmids coding for *trwB* mutations pSU4632 [19], pDEL10 [20]; and in the suicide plasmid pR6K::*oriT*_*P*_ *oriT*_*W*_ [21], carrying the *oriTs* of plasmids R388 and RP4 [22]. Although they are independent constructs, all of them had acquired the same increase in size. No such increase was ever observed in the vector alone or in many other constructs using the pSU vector backbone.

We delimited the region containing the extra DNA by restriction analysis and found that it was always the same region of the vector backbone. We determined the DNA sequence from the vector DNA until the junction with the foreign DNA, in four plasmid constructs that had gained the extra DNA independently during their manipulation. These plasmids were the recombination substrate pRec2*oriT*-Cm [21], pSU4632, pDEL10, and the suicide plasmid pR6K::*oriT*_*P*_ *oriT*_*W*_ integrated into a chromosomal *oriT* copy [22]. The four of them carry different DNA segments cloned in vectors pSU19, pSU24, and pSW23.

**Fig 2:**
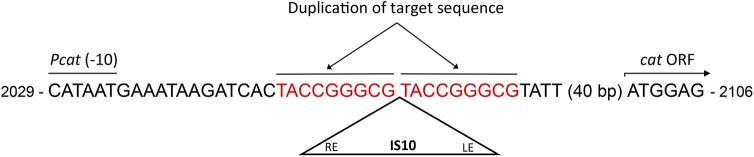
New IS10 target site located upstream of the *cat* ORF. The target site is shown in red, duplicated after IS10 transposition. The insertion site is located 22 bp downstream the -10 element of the promoter and 53 bp upstream the ATG of *cat* ORF. Coordinates correspond to the DNA sequence of pSU8 vector (accession number X53939). IS10 insertion sequence begins in nucleotide 1329 of its DNA sequence (accession number J01829). *Pcat*, *cat* promoter.

Fig 2 shows the results obtained. The site of insertion was located 22 bp downstream the -10 element of the promoter and 53 bp upstream the *cat* ORF, thus blocking the expression of the gene from its cognate promoter. The inserted DNA was identical to the published sequence of insertion sequence IS10 (GenBank Accession number J01829). The junction with the vector DNA occurred exactly at the IS right end, and subsequent sequencing to the IS left end confirmed the precise IS10 transposition event, including the 9 bp duplication of the target DNA (Fig 2). Since all independently obtained integration events occur exactly at the same nucleotide, this DNA sequence represents a previously unreported target for IS10 transposition. The target sequence was 5’-TACCGGGCG-3’, which does not correspond to the consensus target site originally described for IS10 (5’-NGCTNAGCN-3’) [23]. However, IS10 transposase is known to act also on other DNA sequences. Kovarik and collaborators defined a new consensus sequence based on analysis of a large number of IS10 insertions: 5'-NPuCNN-NGPyN-3' [10]. Our target sequence matches this consensus.

### Insertion of IS10 is promoted at high chloramphenicol concentrations

As explained above, IS10 insertions were detected only in a subset of pSU- and pSW-based constructs. A plausible explanation for these results is that the insertion events were selected because of the Cm selection applied. It has been reported previously that IS10 carries outward-facing promoters at both ends [24, 25] and IS10 insertion events that confer resistance to fluoroquinolones by transcriptional activation of adjacent genes in *Salmonella enterica* have been previously reported [26]. So, transcription of *cat* from the IS10 promoter facing outwards could contribute to Cm resistance under conditions in which the intrinsic CAT levels would be insufficient to cope with the Cm selection applied.

**Fig 3:**
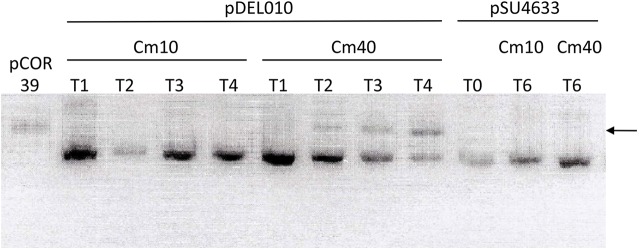
Size of several pSU19-based plasmid constructs after growth under different Cm concentrations. An agarose gel is depicted. The arrow indicates the molecular size after IS10 insertion. T0, DNA used to transform cells at the beginning of the assay; T1, T2, T3, T4, and T6 refer to DNA obtained from cultures of different days. Cm concentration used is indicated in μg/ml, as Cm10 or Cm40.

In order to test this hypothesis, we have grown *E*. *coli* strain DH5α containing pSU19-derived constructs for up to 120 generations in the presence of different concentrations of Cm. Plasmids used were pDEL10, pCOR39, and pSU4633, three pSU19::*trwB* constructs. pSU4633 encodes wild-type TrwB; pDEL10 codes for TrwB variant K275A; as pDEL10 had been observed to change in size before, pCOR39 was obtained from incubation of plasmid pDEL10 until the increase size appeared, and it was used as a control of the size after IS10 insertion. Plasmid pSU4633, not previously shown to change in size after extensive manipulation, was used as a control of size stability. At the end of the experiment, plasmid DNA was extracted and aliquots were run on an agarose gel to compare sizes (Fig 3). Plasmid pSU4633 remained in its original size at the end of the assay, independently of the Cm concentration applied. In contrast, plasmid pDEL10 remained stable growing the host strain in LB supplemented with 10 μg/ml of Cm but acquired IS10 insertion upon growth under 40 μg/ml of Cm.

### Insertion of IS10 permits selection of chromosomal integration events at high chloramphenicol concentrations

The R388 relaxase TrwC pilots the DNA into a recipient bacterium during conjugation and, as a result of its integrase activity, it can catalyze the integration of the incoming plasmid into an *oriT* copy present in the recipient genome ([22]). TrwC-mediated chromosomal integration events described by Agúndez and co-workers [22] took place after mobilization of suicide plasmid pR6K::*oriT*_*P*_ *oriT*_*W*_, which was constructed using pSW23 backbone, into an *E*. *coli* strain carrying a chromosomal copy of R388 *oriT* in place of the *lacZ* gene. Integrants were selected with Cm 25 μg/ml and integration events were checked by PCR analysis with primers amplifying a region of 1 kb of the expected cointegrate molecule. However, the authors observed sometimes an amplicon of 2 kb, suggesting again IS10 integration in a pSU-derived vector, so we decided to characterize these insertion events. We confirmed by DNA sequencing of the PCR product that the bigger amplicons had an IS10 insertion in the same target sequence located upstream of the *cat* gene.

**Fig 4:**
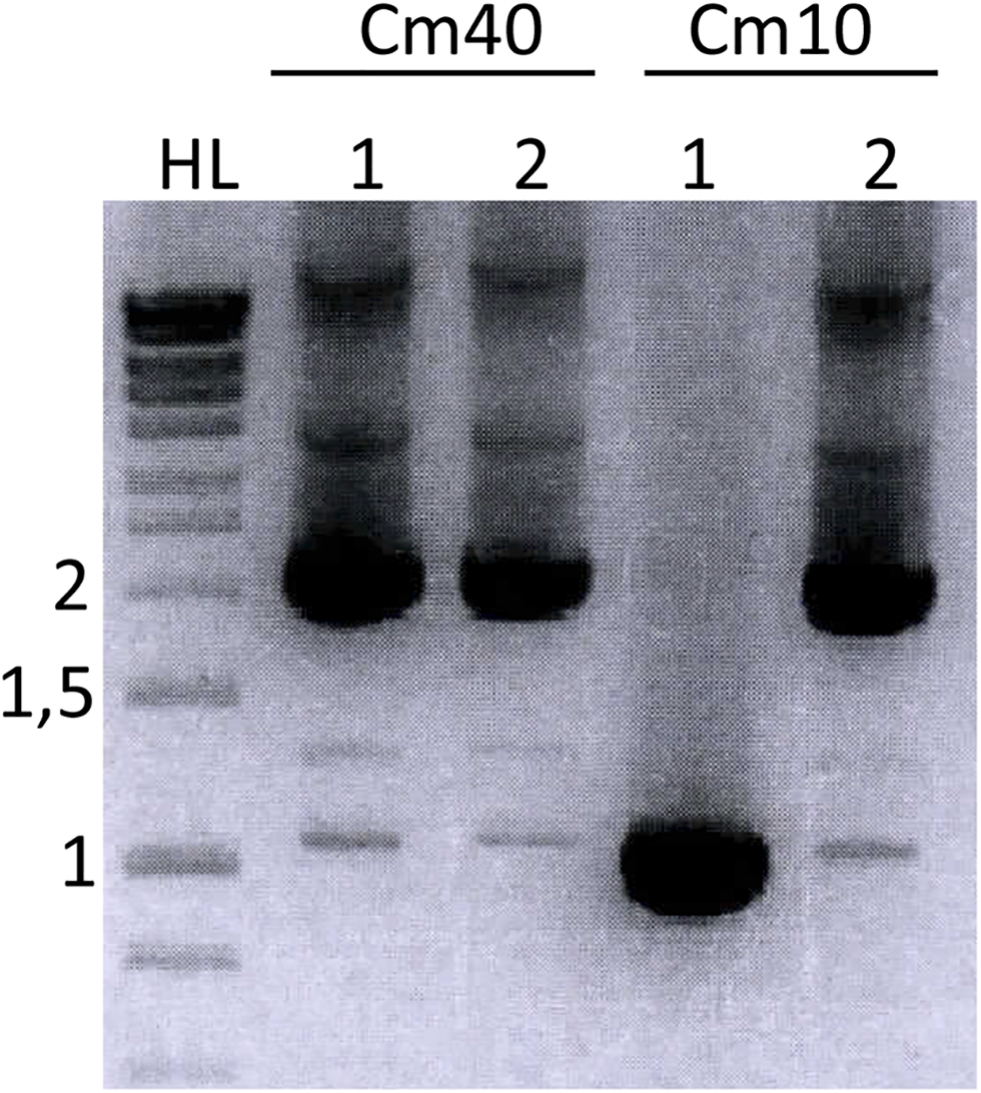
PCR analysis of chromosomal integration events. 1 and 2 indicate two different colonies obtained under each condition tested. Cm40/10 refers to the concentration of Cm (in μg/ml) used for selecting integration events. HL, Hyperladder I (Bioline), with the size of relevant bands indicated in kb on the left.

To elucidate if the pressure exerted by the Cm selection was responsible for the detection of IS10 insertion events, we tested chromosomal integration selecting integrants in both 10 and 40 μg/ml of Cm (Fig 4). While two thirds of the integrants selected under low antibiotic pressure were found to have IS10 insertion, 100% of the integrants obtained with Cm40 selection had acquired it (mean of at least 3 independent experiments, with 10 colonies analysed in each experiment), suggesting that IS10 insertion is necessary for the integrants to survive under the higher Cm selection applied.

## Discussion

In this work we describe a new target sequence for IS10 transposition present in the commonly used pSU and pSW families of cloning vectors. Although transposon Tn10 is not present in the originally sequenced genome of *E*. *coli*, IS10 element is present in 16% of *E*. *coli* and *Shigella* genomes in GenBank [27]. It was described that IS10 has a marked preference for insertion in the consensus site 5’-NGCTNAGCN-3’, causing a 9-bp duplication [23]. However, there are previous works that report insertion in target sequences out of the consensus, and in fact, Kovarik and co-workers redefined the IS10 consensus site to 5'-NPuCNN-NGPyN-3' after analysis of a large collection of IS10 transposition events. The target site reported here matches with this newly defined consensus.

We have found IS10 insertion upstream the *cat* ORF in different independently constructed plasmids based on the pSU backbone, in completely different experiments. The results indicate that transposition events are selected upon high Cm selection. The most surprising result is the occurrence of this phenomenon only in certain plasmid constructs, and not in others. pSU4633 is identical to pDEL10 and pSU4632, only the two latter code for different point mutations in *trwB*, coding for TrwB variants K275A and K136T. TrwB K275A affects transfer of both DNA and protein during bacterial conjugation [20], and a recent study has determined that it affects the ATPase activity of the protein ([28]; Larrea *et al*., submitted for publication); TrwB K136T is an ATPase-deficient, transfer-deficient protein previously characterized [19]. IS10 insertions were commonly detected in the two plasmids carrying *trwB* mutations, while the plasmid coding for wild-type TrwB was never observed to change in size, not even after prolonged incubation with high Cm concentrations (Fig 4). The other plasmids frequently found to acquire IS10 all have two *oriT* copies repeated in tandem.

It must be noted that no conjugative machinery is present in the experiments performed to select IS10 transposition events (Fig 3), which occur solely after incubation of the plasmids in host cells not carrying any other element of the conjugative apparatus. Thus, an effect due to conjugative DNA transfer intermediates can be discarded. The most plausible explanation is that the DNA segment cloned in these plasmids could either confer instability to the cell, or affect plasmid stability, leading to low levels of CAT. Insertions lying in the promoter of the chloramphenicol resistant gene would lead to higher *cat* expression levels, which could compensate the slower metabolism or lower number of plasmid molecules coding for the gene. This idea is supported by the observation that other plasmids based on the same family of vectors do not suffer this phenomenon under the same laboratory conditions. In fact, pSU4633 and pSU4632 differ only in two base pairs, yet the former remains stable while the latter acquires IS10 after incubation with Cm40 (Fig 3).

The reason why certain TrwB variants and the presence of two *oriTs* in the same molecule could be deleterious to the plasmid or to the cell, remains to be determined. TrwB has DNA binding and ATPase activities, both of which could be toxic if deregulated. Both K136T and K275A variants (coded by plasmids pSU4632 and pDEL010) have been analysed in detail and they are affected in their ATPase activity ([19, 29]; Larrea *et al*., submitted for publication). As for the two *oriT* copies, which are in all cases either identical or highly related, *oriTs* are DNA sequences where many DNA binding proteins are recruited, including host factors; and two copies in tandem may undergo recombination. In summary, they could act as protein recruiting factors that could interfere with normal plasmid replication or transcription processes.

From a practical point of view, our suggestion for the ample scientific community using the pSU/pSW families of cloning vectors would be to use low Cm selection conditions, to avoid selection of IS10 transposition events into their constructs. In fact, transposition activity has been reported to be increased under certain stress conditions, such as long incubation of bacterial cultures in the stationary phase of growth [30] or UV light induction [31]. In a similar way, incubation with high concentrations of Cm could be a stressful situation that increases IS10 transposition.

## Materials and Methods

### Bacterial strains, plasmids, and growth conditions

*Escherichia coli* strain DH5α [32] containing different pSU-derived plasmids was used as host for growth under different Cm concentrations. Strains Π1 [5] and s17.1λ*pir* [33] were used as donor cells for the integration assays, while strains HMS174 [34], CMS1 and CMS2 [22] were used as recipient cells. Bacteria were grown in Luria-Bertani (LB) broth, supplemented with agar for solid culture. For selection, antibiotic were used at the following concentrations: ampicillin, 100 μg/ml; chloramphenicol, 25 μg/ml (except when other concentrations are indicated); erythromycin, 200 μg/ml; rifampicin, 100 μg/ml; kanamycin monosulphate, 50 μg/ml. When using Π1 strain, thymidine was supplemented to a final concentration of 0.3 mM. The plasmids used in the present study are listed in Table 1.

**Table 1:** Plasmids used in this work.

Plasmid Name	Description	Reference
pR6K::oriTP oriTw	pSW23::*oriT*_*P*_+*oriT*_*W*_	[21]
pCIG1028	pSU19::R388 *oriT1-oriT2*	[15]
pCIG1032	As pCIG1028, *oriT1* from pKM101	[15]
pCIG1064	As pCIG1028, F *oriT1-oriT2*	[15]
pCIG1066	As pCIG1028, pKM101 *oriT1-oriT2*	[15]
pCIG1077	pKK223-3::*P*_*ABC*_ *trwA-trwL*	[35]
pCOR39	pDEL10::*IS10*	This work
pDEL10	pSU24::*trwB(K275A)*	[20]
pRec2*oriT*-Cm	pSU19:: R388 *oriT1-oriT2*	[21]
pSU19	Cloning vector	[3]
pSU4632	pSU24::*trwAtrwB(K136T)*	[19]
pSU4633	pSU24::*trwAtrwB*	[19]

### Growth under different Cm concentrations

Cm concentrations assayed were 10, 25, and 40 μg/ml. Single colonies were grown overnight (approximately 20 generations) on LB with the corresponding amount of Cm, then 10 μl were diluted into 10 ml fresh medium for further growth and the rest of the grown culture was used for DNA analysis. This cycle was repeated for 6 consecutive days.

### Plasmid DNA extraction and analysis

Plasmid DNA was extracted with the GenElute Plasmid Miniprep Kit (Sigma). DNA was analyzed after incubation with restriction endonucleases (Thermo Scientific) in agarose gels. DNA sequences were determined by Macrogen Inc. DNA Sequencing Service (Amsterdam, The Netherlands). Sequencing of pSU-derived plasmids was carried out using oligonucleotide 5´-TTGGCGAAAATGAGACGTTG-3’, binding 121 nt upstream of the *cat* gene.

### Chromosomal integration assays at different chloramphenicol concentrations

Integration assays were performed as described in [22]. Briefly, the Cm-resistant pR6K::*oriT*_*P*_ *oriT*_*W*_ suicide plasmid that is able to replicate in the donor but not in the recipient is mobilized from Π1 to HMS174, CMS1 or CMS2 strains. Donor Π1 also contains pCIG1077 [35], a helper R388Δ*oriT*. CMS1 and CMS2 are isogenic to HMS174, except that they contain a chromosomal copy of the *oriT* in place of the *lacZ* gene, with the *nic* site lying in the lagging and leading strands, respectively. After mobilization of pR6K::*oriT*_*P*_ *oriT*_*W*_, integrants are selected in Cm, since the recipient cells become Cm^R^ only after integration of the suicide plasmid. Integrants were selected in LB plates containing 10 or 40 μg/ml of Cm. Integration is confirmed by PCR using primers P_A_ (5’-ATGACCATGATTACGGATTCA-3´), annealing to the 5´ end of the *lacZ* gene, and P_C_ (5´GCCTCAAAATGTTCTTTACGA-3´), annealing to the 3´end of the *cat* gene, that amplify a specific region of the cointegrate molecule [22]. DNA sequence of the amplified band was determined with oligonucleotides P_A_ and P_C_.
